# The impact of population dynamics on the population HIV care cascade: results from the ANRS 12249 Treatment as Prevention trial in rural KwaZulu‐Natal (South Africa)

**DOI:** 10.1002/jia2.25128

**Published:** 2018-07-19

**Authors:** Joseph Larmarange, Mamadou Hassimiou Diallo, Nuala McGrath, Collins Iwuji, Mélanie Plazy, Rodolphe Thiébaut, Frank Tanser, Till Bärnighausen, Deenan Pillay, François Dabis, Joanna Orne‐Gliemann, the ANRS 12249 TasP Study Group

**Affiliations:** ^1^ Centre Population et Développement Institut de Recherche pour le Développement Université Paris Descartes Inserm Paris France; ^2^ Africa Health Research Institute KwaZulu‐Natal South Africa; ^3^ School of Nursing and Public Health Africa Health Research Institute University of KwaZulu‐Natal KwaZulu‐Natal South Africa; ^4^ Faculty of Medicine and Faculty of Social, Human and Mathematical Sciences University of Southampton Southampton UK; ^5^ Research Department of Infection and Population Health University College London London UK; ^6^ Department of Global Health & Infection Brighton and Sussex Medical School Falmer Brighton UK; ^7^ ISPED Inserm Bordeaux Population Health Research Center Université de Bordeaux Bordeaux France; ^8^ Department of Global Health & Population Harvard School of Public Health Harvard University Boston MA USA; ^9^ Institute of Public Health Faculty of Medicine Heidelberg University Heidelberg Germany; ^10^ Division of Infection and Immunity University College London London UK

**Keywords:** HIV care continuum, Public health, Structural drivers, Migration, Cross‐sectional cascade, Rural South Africa, Population dynamics

## Abstract

**Introduction:**

The universal test and treat strategy (UTT) was developed to maximize the proportion of all HIV‐positive individuals on antiretroviral treatment (ART) and virally suppressed, assuming that it will lead to a reduction in HIV incidence at the population level. The evolution over time of the cross‐sectional HIV care cascade is determined by individual longitudinal trajectories through the HIV care continuum and underlying population dynamics. The purpose of this paper is to quantify the contribution of each component of population change (in‐ and out‐migration, HIV seroconversion, ageing into the cohort and definitive exit such as death) on the HIV care cascade in the context of the ANRS 12249 Treatment as Prevention (TasP) cluster‐randomized trial, investigating UTT in rural KwaZulu‐Natal, South Africa, between 2012 and 2016.

**Methods:**

HIV test results and information on clinic visits, ART prescriptions, viral load and CD4 count, migration and deaths were used to calculate residency status, HIV status and HIV care status for each individual on a daily basis. Position within the HIV care continuum was considered as a score ranging from 0 (undiagnosed) to 4 (virally suppressed). We compared the cascade score of each individual joining or leaving the population of resident adults living with HIV with the average score of their cluster at the time of entry or exit. Then, we computed the contribution of each entry or exit on the average cascade score and their annualized total contribution, by component of change.

**Results:**

While the average cascade score increased over time in all clusters, that increase was constrained by population dynamics. Permanent exits and ageing into the people living with HIV cohort had a marginal effect. Both in‐migrants and out‐migrants were less likely to be retained at each step of the HIV care continuum. However, their overall impact on the cross‐sectional cascade was limited as the effect of in‐ and out‐migration balanced each other. The contribution of HIV seroconversions was negative in all clusters.

**Conclusions:**

In a context of high HIV incidence, the continuous flow of newly infected individuals slows down the efforts to increase ART coverage and population viral suppression, ultimately attenuating any population‐level impact on HIV incidence.

**Clinical Trial Number:**

NCT01509508 (clinicalTrials.gov)/DOH‐27‐0512‐3974 (South African National Clinical Trials Register).

## 1 Introduction

Early antiretroviral treatment (ART) of HIV‐positive patients has been shown to prevent transmission of HIV 1, in addition to individual benefits in terms of reducing morbidity and mortality 2, 3. The universal test and treat (UTT) strategy was developed by extending this idea to the population level under the hypothesis that HIV testing of all adult members of a community, followed by immediate ART initiation of nearly all HIV‐positive individuals, regardless of immunological or clinical staging, will prevent onward transmission and reduce HIV incidence in the community. This strategy is supported by modelling work suggesting that such an approach, if successfully implemented, could eliminate HIV transmission in South Africa 4, and in a large population‐based cohort from rural KwaZulu‐Natal, South Africa, demonstrating a strong inverse association between ART coverage and HIV acquisition 5.

The implementation of any UTT strategy involves improving all steps of the “cascade of HIV care” 6, 7, as set out by UNAIDS in their 90‐90‐90 targets to be reached by 2020, that is 90% of all people living with HIV (PLWHIV) being diagnosed, 90% of those being in care and receiving ART and 90% of those on ART with viral suppression 8. This type of HIV care cascade is measured by a population‐based and cross‐sectional set of indicators estimating the proportion of HIV‐positive individuals diagnosed, in care, on ART and virally suppressed among all PLWHIV residing within a certain geographical area at a specific time point, although alternative longitudinal measurements of the cascade exist 9.

The evolution over time of a cross‐sectional HIV care cascade is determined by two elements: (i) the journey of HIV‐positive individuals through the care continuum (longitudinal care trajectories), and (ii) the changes in the underlying population of resident PLWHIV (population dynamics, i.e. in‐ and out‐migration, HIV seroconversion, ageing into the cohort and definitive exit such as death).

Migration is one component of population dynamics. It has been shown in rural South Africa that even relatively short‐distance migration events confer substantial additional risk of HIV acquisition 10. More generally, mobility is associated with increased risk of ART non‐adherence, lost to follow‐up, deterioration in CD4 count, HIV‐related death, development of drug resistance and general non‐continuity of HIV care 11.

Beyond the understanding of individual trajectories through the HIV care continuum (sometimes referred as longitudinal cascade 12), it is also important to quantify the structural effect of the dynamics of the PLWHIV population and its impact on the cross‐sectional HIV care cascade.

The ANRS 12249 Treatment as Prevention (TasP) trial implemented a UTT strategy in rural KwaZulu‐Natal to test the impact of universal ART on population HIV incidence. The objectives of this analysis are: (i) to document the dynamics of the PLWHIV population in the trial area over time, distinguishing the different components of population change; (ii) to identify the position within the HIV care continuum of individuals joining or leaving the PLWHIV population at the time of entry or exit; (iii) to compare their care position with the average care position of the local PLWHIV population; and finally, (iv) to quantify the contribution of each component of population change on the cross‐sectional HIV care cascade.

## 2 Methods

### 2.1 Study setting and design

The TasP trial was a phased two‐arm cluster‐randomized trial implemented by the Africa Health Research Institute (AHRI) in Hlabisa sub‐district, northeast KwaZulu‐Natal, South Africa, in a rural area with approximately 28,000 isiZulu‐speaking resident adults. Adult HIV prevalence in the sub‐district was around 30% 13, 14. Hlabisa sub‐district is characterized by frequent migration 15, 16, low marital rates and late marriage 17. On average, one adult in ten in the trial area is employed 18.

The trial aimed to investigate whether immediate ART initiation offered to all HIV‐positive individuals, identified through home‐based HIV testing will reduce HIV incidence in the area. Trial protocol and study procedures have previously been reported in detail 19, 20.

The trial was implemented from March 2012 to June 2016 using a phased approach: four clusters opened in 2012, six additional clusters opened in 2013 and 12 in 2014. All 22 clusters (2 × 11) were followed until mid‐2016. Each cluster was designed to correspond to an average of about 1,000 resident adults.

The UTT strategy tested in TasP trial had two main components: (i) repeat home‐based HIV testing (both arms) and (ii) immediate ART initiation (intervention arm).

In both trial arms, HIV counsellors visited all local households and enumerated, with the household head or another adult household member, all resident adult (16 years and above) household members (initial census during the first survey round). Residency was defined in trial protocol as spending at least four nights per week within the homestead (*de jure* household members at the survey date). At each subsequent home‐based survey round, conducted six‐monthly, newly identified households and all previously registered households were (re)visited and the list of resident adult household members was updated. Exits (including deaths and out‐migration from trial area) were documented as reported from another household member.

Eligible individuals providing written informed consent in isiZulu responded to a socio‐demographic and sexual behaviour questionnaire and gave a finger prick sample collected as a dried blood spot (DBS), used for HIV incidence estimation. HIV counsellors also offered individuals point‐of‐care rapid HIV counselling and testing. Participants identified HIV‐positive through DBS but who refused HIV rapid test at a specific survey round were not notified of their DBS result. However, they were re‐invited to test for HIV in a subsequent survey round. All trial participants identified as HIV positive (through rapid HIV test or self‐report) were referred to a local trial clinic set up by the trial and situated in the trial cluster in which they lived, located at less than 45 minutes walking distance. From May 2013, support for linkage to trial clinics through phone calls and home visits by a dedicated trial team was offered to individuals not linked to care within three months after referral.

In the trial clinics of the control clusters, HIV‐positive adults were offered ART according to national guidelines (initially CD4 count ≤350/μL, then ≤500/μL from January 2015). In the trial clinics of the intervention clusters, all HIV‐positive adults were offered the opportunity to begin ART immediately regardless of CD4 count or clinical staging. The trial area was also served by three local governmental primary care clinics of the department of health providing HIV testing, HIV care and ART according to national guidelines only 21. HIV‐positive participants of both arms could opt to receive HIV care in primary care clinics or transfer to a trial clinic.

The Biomedical Research Ethics Committee (BREC), University of KwaZulu‐Natal, South Africa (BFC 104/11) and the Medicines Control Council of South Africa approved the trial. The trial was also registered on ClinicalTrials.gov: NCT01509508 and South African National Clinical Trials Register: DOH‐27‐0512‐3974.

### 2.2 Sources of data

The main data source for this analysis was the trial database, which provided information on trial registrations and trial exits; uptake and results of home‐based rapid HIV testing; third generation ELISA HIV serological results from DBS; and clinic visits, ART prescription and viral loads of PLWHIV seen in trial clinics.

Two additional data sources were used to capture information from PLWHIV seen in local governmental clinics: (a) viral loads and CD4 counts from National Health Laboratory Service (NHLS); and (b) ART clinic visits and ART prescriptions from the AHRI clinical database (ACCDB) which is managed by the Hlabisa Department of Health and AHRI. Both NHLS and the ACCDB database contain data from Hlabisa primary care clinics since 2004 21. Linkage between trial, NHLS and ACCDB database used a probabilistic score based on first name, last name, date of birth, South African ID number and cell phone number. Matching of the databases was approved by the BREC in March 2013 (Protocol Amendment 4).

### 2.3 Daily statuses

Residency, HIV status and HIV care status (if resident and HIV‐positive) were estimated daily for all individuals registered within the trial.

Out‐migration and permanent exits were documented through trial exits. In‐migration and ageing into the cohort (16th birthdays) were derived from the resident household members lists updated at every round. Dates of in‐migration events were randomly imputed using a random point approach (uniform distribution) between the last home visit where individuals were known as non‐resident and the first home visit where they were considered as resident. An individual could contribute several migration events, for example if he/she out‐migrated from the trial area and re‐entered the trial area at a later date.

HIV status was identified using multiple sources: repeat DBS, repeat rapid tests, HIV‐positive self‐reports and HIV clinic visits in trial and/or local governmental clinics, providing information on HIV status at specific dates. A case‐by‐case investigation (including additional laboratory analysis) was performed to solve any inconsistent data. An individual was considered as HIV‐negative before the last known negative status and as HIV‐positive after the first known positive status. For those in whom a negative status was followed by a positive one, date of seroconversion was imputed using a random point approach (uniform distribution). For individuals entering the trial cohort, the first opportunity for the trial team to ascertain their HIV status occurred *de facto* after their entry. For some, a previous record was found in NHLS and/or in ACCDB database. For the others, we imputed if and when a potential unobserved HIV seroconversion occurred using the observed incidence within the same cluster and for people of the same sex. A similar approach was used to impute a possible seroconversion before the end of trial follow‐up for those whose last observed HIV status was negative, assuming they remained undiagnosed until the end of trial follow‐up. Individuals with no observed HIV status (i.e. with no data on HIV status) were excluded from the analysis.

HIV care statuses were defined as: (i) undiagnosed; (ii) diagnosed but not actively in care (i.e. never in care or lost to follow‐up); (iii) actively in care but not on ART; (iv) on ART but not virally suppressed (undocumented viral load or viral load over 400 copies/μL); and (v) in care, on ART and documented viral suppression. Position within the HIV care continuum was considered as a score ranging from 0 (undiagnosed) to 4 (virally suppressed). We note *S*_*i,t*_ the score of an individual *i* at time *t*.

An individual was considered as *being diagnosed* if he/she had at least one positive rapid test, one self‐report as HIV‐positive or had visited a primary care clinic. Date of HIV diagnosis was defined as the date of home‐based HIV testing for individuals newly diagnosed by the trial counsellors. For those already diagnosed when they were interviewed, we considered the date of the first record in NHLS or ACCDB database, if any. It should be noted that for individuals tested in primary care clinics, a CD4 count is performed the same day in case of positive result, resulting in a record in NHLS database. For the few individuals self‐reporting being HIV‐positive but with no previous record in NHLS/ACCDB, we considered as a proxy of the diagnosis date the date of the self‐report.

*Being actively in HIV care* in a trial clinic was defined as not being >90 days late of a scheduled clinic appointment date 22 (visits were scheduled monthly if on ART, six‐monthly if not yet eligible for ART in control arm). Due to the nature of the data available in NHLS and ACCDB database with neither database being exhaustive (some individuals recorded in one database were not found in the other, in particular pre‐ART patients not covered by ACCDB), we were not able to use the same definitions regarding being actively in HIV care in primary care clinic. For patients matched with ACCDB database, being actively in HIV care was defined as having a clinic visit recorded in the last four months (one month for next appointment, this database being limited to ART patients supposed to visit clinics monthly, +3 months late). For patients matched with NHLS database (which contains only laboratory test results), it was having a CD4 count or a viral load recorded in the previous 13 months, CD4 count and viral load data being considered a proxy for clinic visits, following the approach proposed by Lessel et al. 23.

*Being on ART* was defined as having an ART prescription recorded in the trial or the ACCDB database in the previous 3 months or as having an undetectable viral load (<400 copies/μL) recorded in the last 13 months in the NHLS database, an undetectable viral load being considered here as a proxy for being on ART for HIV patients recorded in NHLS database but not found in ACCDB.

*Viral suppression* was defined as a viral load fewer than 400 copies/μL. The viral load at a given date was estimated by linear interpolation using all results recorded in the trial and the NHLS database. The viral load was considered as undocumented before the first available record.

### 2.4 Components of population change

We broke down change of the resident PLWHIV population into five components: (i) ageing into the cohort; (ii) HIV seroconversion; (iii) in‐migration to the cluster; (iv) out‐migration from the cluster; and (v) permanent exits (deaths or loss of the ability to provide an informed consent, e.g. due to illness). Dates of out‐migration and permanent exit events were collected by a specific form, while dates of in‐migration, ageing into the cohort and HIV seroconversion were estimated (see previous section). A same individual could have experienced several events over the course of the trial.

### 2.5 Statistical analysis

**Figure 1:**
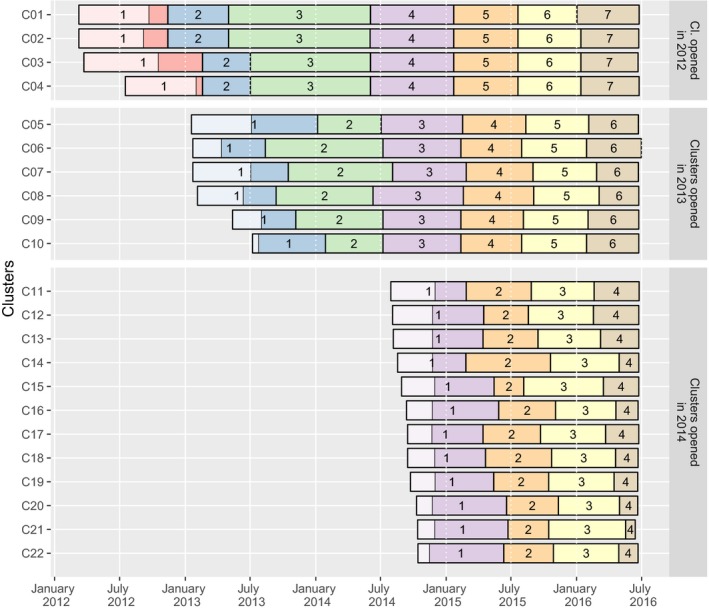
Dates of home‐based survey rounds activities by clusters, ANRS 12249 TasP trial (2012 to 2016). The light areas in round 1 indicate the time required to complete the initial census of the resident population.

As all clusters were not open at the same time, and have therefore different observation periods, we performed the statistical analysis per cluster, from the end of the initial population census to the beginning of the last survey round (Figure 1). In addition, this allows us to see if the impact of population dynamics on the HIV care cascade is uniform or heterogenous between clusters.

For a given cluster *c*, the average cascade score *μ*_*c,t*_ at time *t* is equal to Σ_*i*_ *S*_*i,t*_/*n*_*c,t*_ where *n*_*c,t*_ corresponds to the number of PLWHIV aged 16 years or older, residing within the cluster *c* at the date *t*. This average score is a summary statistic of the cascade distribution.

We computed, per cluster, rates of each population change component from the resident HIV‐positive population per person years of residency.

To compare the cascade score of a specific individual *i* joining or leaving the PLWHIV population with the average score of their cluster at the time *te* of entry/exit, we computed *δ*_*i,te*_ as *S*_*i,te*_ − *μ*_*c,te*_. We used Student's *t*‐test to test if the mean of *δ*_*i,te*_ by type of population movement differed significantly from zero.

The contribution *C*_*e*_ of a specific event *e* on the change in the average cascade score at cluster level depends on the PLWHIV population size and could be defined as *δ*_*i,te*_/*n*_*c,te*_ for an entry event and −*δ*_*i,te*_/*n*_*c,te*_ for an exit event. For a given cluster *c*, the sum of all event contributions, that is *SC*_*e*_ **= Σ_*e*_ *C*_*e*_, provides the total contribution of population change on the average cascade score over time. As all clusters were not observed for the same amount of time, we annualized these total contributions for comparing clusters: *aSC*_*e*_ = *SC*_*e*_/*T*_*c*_, where *T*_*c*_ is the observation period for cluster *c*.

All analyses were performed using R version 3.4.1 24.

## 3 Results

Overall, 28,419 adults were registered over the course of the trial. 338 individuals exited the trial area before the end of the initial census or were registered during the last survey round. Among the remaining 28,081 individuals: HIV status was undocumented for 2,582 (9.2%); and 16,994 (60.5%) remained HIV negative over the analysis period; thus, 8,505 individuals were part of the resident population of PLWHIV over the analysis period and included in the analysis.

**Figure 2:**
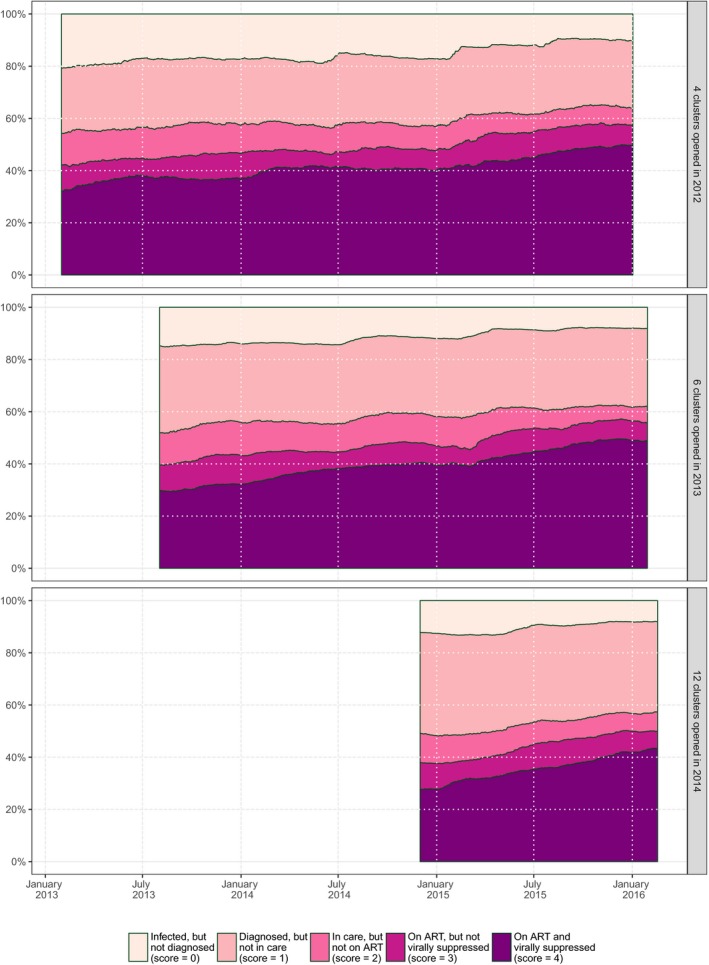
Evolution over time of the population cross‐sectional HIV care cascade per group of clusters, ANRS 12249 TasP trial (2012 to 2016).

**Figure 3:**
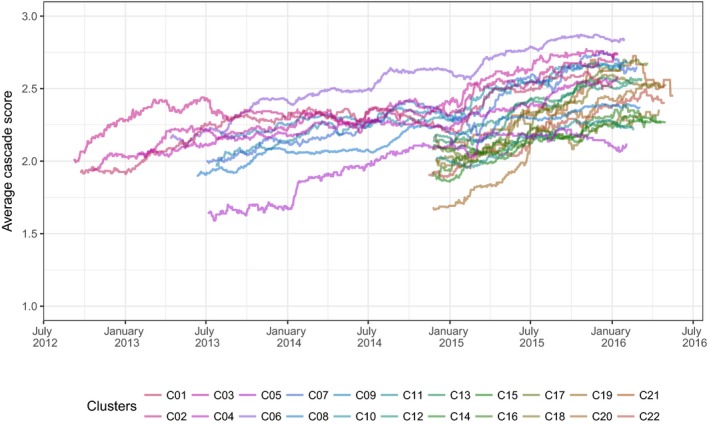
Trends of the average cascade score per cluster over time, ANRS 12249 TasP trial (2012 to 2016). Each line represents a different cluster. All clusters did not open at the same time (cf. Figure 1): 4 clusters opened in 2012, 6 in 2013 and 12 in 2014.

The population HIV care cascade improved over time: the proportion of PLWHIV in care, on ART and virally suppressed increased from approximately 30% to 45% to 50%, while the proportion of PLWHIV not in care (diagnosed or undiagnosed) decreased from approximately 50% to 35% to 40%, depending on the duration of trial follow‐up in each cluster (Figure 2). Although the level of the average cascade score differs between clusters, trends present a similar pattern of increase in all clusters (Figure 3).

**Figure 4:**
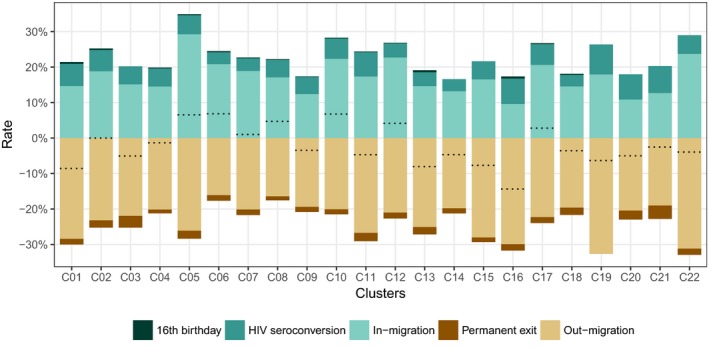
Annual growth rates of the resident PLWHIV population, by population change components and per cluster, ANRS 12249 TasP trial (2012 to 2016). Dotted lines indicate the sum of all rates, i.e. net annual population growth rate.

The PLWHIV population is characterized by a high turnover over the study period: the overall annual exit rate is 22.6% (2,979 exits for 13,180 person years) and the overall annual entry rate 22.4% (2,948 entries for 13,180 person years). Population dynamics were mainly due to out‐ and in‐migration (annual rate, respectively, of 21.0% and 17.3%). New HIV infections accounted for a 4.8% annual increase in the PLWHIV population (625 events: 510 observed seroconversions and eight estimated unobserved seroconversions before the first observed positive status and 117 after the last negative one). The annual rate of permanent exits was 1.6% (216 events: 186 deaths and 30 individuals who lost their ability to consent). Finally, we observed 29 participants already HIV positive when they reached 16 years of age during the trial. The distribution of population change by component is similar between clusters, although the overall net growth is negative for 14 clusters and positive for the other 8 (Figure 4). Basic socio‐demographic characteristics of PLWHIV entering/exiting the resident population are provided in supplementary materials (Table S1).

The average cascade score by cluster could be considered as a good summary statistic of the cross‐sectional cascade as it is highly and significantly (*p* < 0.0001) correlated (Figure S2) with the proportions of diagnosed individuals (score ≥ 1), individuals in care (score ≥ 2), individuals on ART (score ≥ 3), and individuals virally suppressed (score = 4).

**Figure 5:**
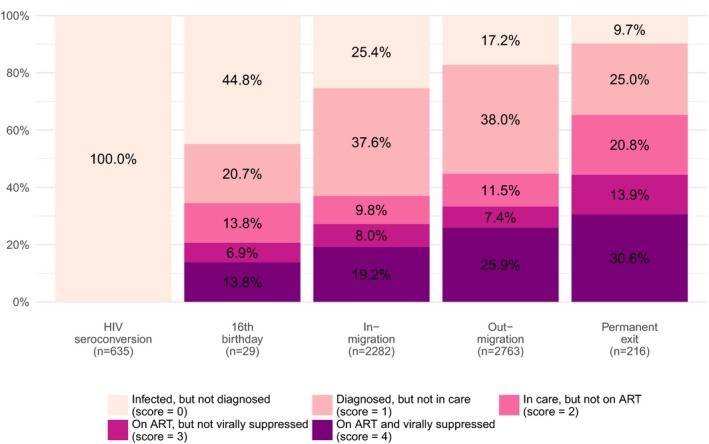
Position within the HIV care continuum at the date of entry/exit, by population change component, ANRS 12249 TasP trial (2012 to 2016).

**Figure 6:**
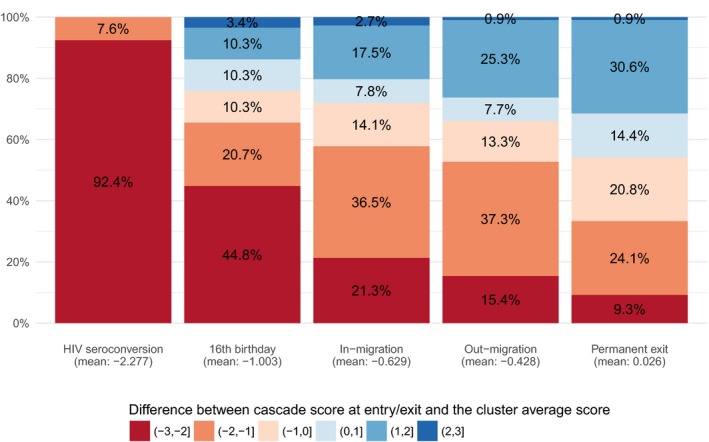
Distribution of the differences between HIV care cascade score at entry/exit and the cluster average score at the same date, by population change component, ANRS 12249 TasP trial (2012 to 2016).

HIV seroconverters, who were undiagnosed at the date of seroconversion (Figure 5), had a lower cascade score compared to PLWHIV in the same cluster and at the same date (mean difference: −2.278, *p* < 0.0001, Figure 6). Half of the young people turning 16 years old and already infected by HIV were undiagnosed and only a fifth were on ART at that time (mean difference: −1.039, *p* = 0.0007). Permanent exits had on average a similar score to the rest of PLWHIV in their cluster (mean difference: 0.030, *p* = 0.7541), two‐thirds being actively in care at the date of the exit. Most migrants had a lower position compared to their cluster population, the difference being higher for in‐migrants (mean difference: −0.630, *p* < 0.0001) than out‐migrants (−0.429, *p* < 0.0001): 26% of out‐migrants were on ART and virally suppressed at the date they out‐migrated compared to 19% among in‐migrants at arrival.

**Figure 7:**
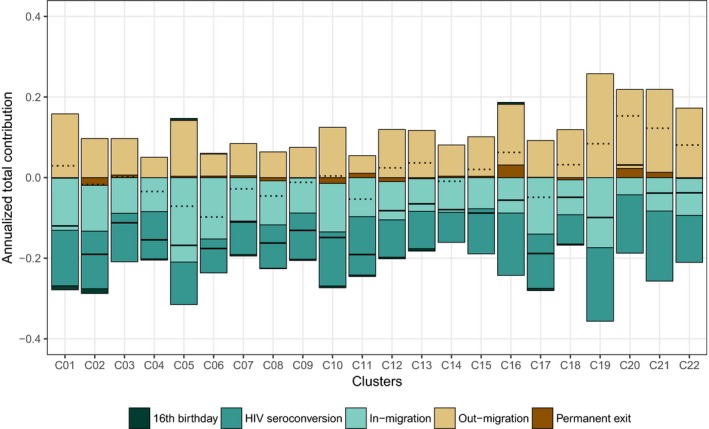
Annualized total contribution of population change on cluster average cascade score, by component of population change and per cluster, ANRS 12249 TasP trial (2012 to 2016). Dotted lines indicate the sum of the total contribution of in‐ and out‐migration. Black lines indicate the sum of total contribution of all events. Examples of reading: in cluster C01, in‐migration events reduced annually the cluster average cascade score by 0.127 while out‐migration events increased it by 0.161. Therefore, the overall contribution of migration is +0.034 per year.

In all clusters (Figure 7), in‐migration contributed negatively to the cluster average cascade score (from −0.210 to −0.043, median: −0.093) while out‐migration contributed positively (from +0.046 to +0.255, median: +0.108). Because out‐migrants had a lower score compared to local HIV residents, when they left their cluster, it mechanically increased the average cascade score of the population left behind. In‐ and out‐migration compensated each other: the total contribution of migration was negative in 10 clusters and positive in the other 12, and close to zero in several clusters (from −0.099 to +0.154, median: +0.002). The contribution of permanent exits (from −0.019 to +0.032, median: +0.001) and 16th birthdays (from −0.012 to +0.005, median: −0.004) was marginal with no clear pattern between clusters. HIV seroconversion had a negative contribution to the cascade in all clusters (from −0.182 to −0.072, median: −0.117), resulting in a total contribution of all events being negative almost everywhere (from −0.190 to +0.032, median: −0.112).

The imputation of potential unobserved HIV seroconversions had a marginal effect. Re‐analysing the data without considering imputed seroconversions (Figure S3) did not change the results substantively, and the annualized total contribution of all components on the cluster average score ranged from −0.174 to +0.069 (median: −0.098).

## 4 Discussion

Our results demonstrate that the turnover of the PLWHIV population residing in our trial area was high, more than one‐fifth being replaced every year. While the average HIV care cascade score increased over time in all clusters, that increase was limited due to population dynamics, the total contribution of all entries and exits being systematically negative, and it was mainly due to new HIV infections.

In‐migrants joined the PLWHIV population at earlier stages of the cascade and lowered the cluster average score. Even if out‐migrants usually left at a slightly higher position in the cascade than in‐migrants when they arrived, there were also at earlier stages compared to the rest of the resident population. Although this could be counterintuitive, at the population level, it had a positive effect on the cluster average score. As a result, out‐ and in‐migration had a balanced impact on the cross‐sectional cascade, both effects cancelling out.

Data collected through the ANRS 12249 TasP trial constitute a unique opportunity to make a fine description of individual trajectories over time through HIV services, on a daily basis, which can be done only in very few settings. However, to fully understand our results, it is crucial to understand the limitations of the data. Due to the nature of available data sources, different definitions and assumptions were required to estimate individual statuses. First, in‐migration events were not directly collected by TasP fieldworkers and have therefore been derived by comparison of resident household members lists between survey rounds. Second, while HIV care statuses were measured precisely within trial clinics, we had to use proxy indicators (based on laboratory data in particular) for being in HIV care and being on ART in local governmental clinics. Measurement of entry into care is generally robust because a CD4 count and/or a viral load is almost associated with first clinic visit. However, the identification of individuals exiting care is delayed for individuals matched only to NHLS database. In addition, considering our matching algorithm, the probability that a trial participant was wrongly associated with a record in ACCDB or NHLS (type‐I error) is relatively small. However, some patients who resided in the trial area could not be matched (type‐II error), for reasons such as data entry errors or use of different names in different settings 25. Third, we did not have any data on individuals receiving HIV care in the private sector or in primary care clinics located outside the Hlabisa sub‐district, potentially resulting in an underestimation of population progression through the HIV care cascade. Our estimates of the proportion of PLWHIV being in care should be considered as lower bounds. Finally, 9.5% of trial participants had an undocumented HIV status (because the fieldworkers were not able to contact them at least once or because they systematically refused rapid HIV tests and to provide a DBS) and were excluded from the analysis. A sensitivity analysis (unpublished yet) suggests that it is not affecting the observed trends of the population HIV care cascade. However, we could expect higher migration rates and a higher HIV incidence in that group. Therefore, our estimates of the contribution of population change on the cascade could thus be seen as conservative estimates.

The ANRS 12249 TasP trial did not demonstrate a significant difference in cumulative incidence by arms 18, due in particular to a low level of linkage to care 26, resulting in a limited increase of ART coverage over time and the absence of differentiation between arms. At the beginning of the trial, population ART coverage among all HIV‐positive adults living in the study area was estimated at 29.6% in intervention arm and 33.7% in control arm. ART coverage rose to 53.4% (+23.8) in the intervention arm and 52.8% (+19.1) in the control arm by 1 January 2016, with the difference between arms not statistically significant (*p* = 0.67) 18.

In our context of high HIV incidence, delays in HIV diagnosis is a key barrier to progress through the population HIV care cascade. Within the ANRS 12249 TasP trial, one‐third of individuals who seroconverted remained undiagnosed after one year, one‐third discovered their HIV‐positive status but did not enter care and one‐third linked to an HIV clinic 27. Overall, with only 17% initiating ART within 12 months after seroconversion, we are far from the 81% expected by the model of Granich and colleagues 4 to eliminate HIV transmission in South Africa. The HIV care trajectories were clearly suboptimal in seroconverters despite the introduction of UTT services and a trial environment, contributing to a continuous transmission of HIV within the population.

Migrants in general had a lower position within the continuum of HIV care than the average population: this group needs specific interventions to link and retain into care over time. This very mobile population is not living separately from the rest of the local population. In this rural area with few job opportunities, people come and go and are still part of local sexual networks. In this trial, around 40% of participants reported having a sexual partner located outside of the trial area, some as far as major cities outside of KwaZulu‐Natal 18. In this paper, we did not analyse migration patterns and their associated factors (in particular gender), such topic needed to be explored further. More generally, the dynamics between migration, the HIV epidemic and care trajectories through the health system require additional investigations.

## 5 Conclusions

Migrants face specific vulnerabilities that limit their retention at each step of the HIV care continuum and coordination to facilitate continued access to care when people move should be developed. In a context of high HIV incidence, the continuous flow of newly infected individuals who are less likely to link to HIV care and to initiate ART, slows down efforts to increase overall ART coverage and population viral suppression, ultimately attenuating any population‐level impact on HIV incidence. Identifying specific interventions to reach newly infected people as early as possible is a crucial step on the way towards the end of the epidemic.

## Competing Interests

CI received honoraria for consulting services rendered to Gilead Sciences. All other authors declare that they have no conflicts of interest.

## Authors' Contributions

CI, JOG, FT, DP and FD designed and implemented the ANRS 12249 TasP trial. JL, JOG and NM developed the research question addressed in this paper. JL and MHD did the statistical analysis. JL wrote the first draft of the manuscript with the support of JOG. All authors contributed to the interpretation and presentation of the findings. All authors approved the final version of the manuscript for submission.

## Funding

This trial was sponsored by the French National Agency for AIDS and Viral Hepatitis Research (ANRS; grant number, 2011‐375), and funded by the ANRS, the Deutsche Gesellschaft für Internationale Zusammenarbeit (GIZ; grant number, 81151938), and the Bill & Melinda Gates Foundation through the 3ie Initiative. This trial was done with the support of Merck and Gilead Sciences, which provided the Atripla drug supply. The Africa Health Research Institute, (previously Africa Centre for Population Health, University of KwaZulu‐Natal, South Africa) receives core funding from the Wellcome Trust, which provides the platform for the population‐based and clinic‐based research at AHRI.

## Supporting information


**Table S1.** Socio‐demographic characteristics of individuals who entered or exited the resident PLWHIV population, by population change component, ANRS 12249 TasP trial (2012 to 2016)
**Figure S2.** Correlation between the average cascade score and proportions of all resident PLWHIV being diagnosed, in care, on ART or virally suppressed at cluster level, ANRS 12249 TasP trial (2012 to 2016)
**Figure S3.** Sensitive analysis: comparison of annualized total contribution of population change on cluster average cascade score, by component of population change and per cluster, with **(A)** and without **(B)** imputation of potential unobserved seroconversions.
