# Supplementary material for: The impact of population dynamics on the population HIV care cascade: results from the ANRS 12249 Treatment as Prevention trial in rural KwaZulu‐Natal (South Africa)
Source: J Int AIDS Soc. 2018 Jul 19;21(Suppl Suppl 4):e25128. doi: 10.1002/jia2.25128 (PMC6053480; doi:10.1002/jia2.25128)
Supplement: Supplementary file 1 — Table S1. Socio‐demographic characteristics of individuals who entered or exited the resident PLWHIV population, by population change component, ANRS 12249 TasP trial (2012 to 2016) Figure S2. Correlation between the average cascade score and proportions of all resident PLWHIV being diagnosed, in care, on ART or virally suppressed at cluster level, ANRS 12249 TasP trial (2012 to 2016) Figure S3. Sensitive analysis: comparison of annualized total contribution of population change on cluster average cascade score, by component of population change and per cluster, with (A) and without (B) imputation of potential unobserved seroconversions. [file JIA2-21-e25128-s001.pdf]

SUPPLEMENTARY MATERIAL

The impact of population dynamics on the population HIV care cascade: results from the ANRS 12249 Treatment as Prevention trial in rural KwaZulu-Natal (South Africa)

ANRS 12249 TASP STUDY GROUP

The members of the ANRS 12249 TasP trial group during the course of the study are as follows:

- *Africa Health Research Institute (Previously Africa Centre for Population Health, University of KwaZulu-Natal), South Africa:* Till Bärnighausen, Kobus Herbst, Collins Iwuji, Thembisa Makowa, Kevi Naidu, Marie-Louise Newell, Nonhlanhla Okesola, Tulio de Oliveira, Deenan Pillay, Tamsen Rochat, Frank Tanser, Johannes Viljoen, Thembelile Zuma
- *University of KwaZulu-Natal, School of Nursing and Public Health, Durban, South Africa:* Frank Tanser*;*
- *Nelson R Mandela School of Medicine, College of Health Sciences:* Nuala McGrath
- *ISPED, Centre INSERM U1219 Bordeaux Population Health, Université de Bordeaux, Bordeaux, France:* Eric Balestre, François Dabis, Sophie Karcher, Joanna Orne-Gliemann, Melanie Plazy, Mélanie Prague, Rodolphe Thiébaut, Thierry Tiendrebeogo
- *University College London, Department of Infection and Population Health:* Collins Iwuji, Nuala McGrath; *Division of Infection and Immunity:* Deenan Pillay
- *INSERM, UMR912 SESSTIM, Université Aix Marseille, Marseille, France:* Sylvie Boyer, Hermann Donfouet, Andrea Gosset, Laura March, Camelia Protopopescu, Bruno Spire
- *Service des Maladies Infectieuses Hôpital Universitaire de Geneve, Geneva, Switzerland:* Alexandra Calmy
- *Centre Population et Développement UMR 196, Université Paris Descartes, Institut de Recherche pour le Développement, Paris, France:* Joseph Larmarange, Maxime Inghels, Hassimiou Diallo
- *AP-HP, Virology, Hôpital Pitié-Salpétrière, INSERM-Sorbonne Universités, UPMC Univ Paris 06, UMR_S 1136, Paris, France:* Vincent Calvez, Anne Derache, Anne-Geneviève Marcelin
- *INSERM U1018, CESP, Epidemiology of Occupational and Social Determinants of Health, Villejuif, France:* Rosemary Dray-Spira, France Lert, Kamal El Farouki
- *London School of Hygiene & Tropical Medicine, London, UK:* Richard Lessells
- *Massachusetts General Hospital, Harvard Medical School, Boston, MA, US:* Kenneth Freedberg
- *Futures Group, Johannesburg, South Africa:* John Imrie
- *EA 3620, Université Paris-Descartes, Laboratoire de Virologie, Hôpital Necker-Enfants Malades, AP-HP, Paris, France:* Marie-Laure Chaix
- *Harvard University, Department of Global Health & Population, Harvard School of Public Health, Boston, United States:* Till Bärnighausen
- *Heidelberg University, Institute of Public Health, Faculty of Medicine, Heidelberg, Germany*: Till Bärnighausen
- Department of Global Health and Infection, Brighton and Sussex Medical School, Brighton, United Kingdom (Collins Iwuji)
- *University of Southampton, Faculty of Medicine and Faculty of Human, Social and Mathematical Sciences, Southampton, United Kingdom:* Nuala McGrath;
- *Academic Unit of Primary Care and Population Sciences, and Department of Social Statistics and Demography:* Colin Newell
- *Sponsor representatives:* Brigitte Bazin, Claire Rekacewicz

Table S1. Sociodemographic characteristics of individuals who entered or exited the resident PLWHIV population, by population change component, ANRS 12249 TasP trial (2012-2016). Characteristics are computed at the date of entry/exit and by event. Some individuals could contribute several times in this table if they experienced several events (for example, a seroconverter who out-migrated and in-migrated back at a later date). All Chi-square tests are significant (p < 0.001).

	Entries			Exits	
**Column percentage**	**16th anniversary (n=29)**	**HIV seroconversion (n=635)**	**In-migration (n=2'282)**	**Out-migration (n=2'763)**	**Permanent exit (n=216)**
**Sex**					
Female	75.9	82.4	73.7	74.9	55.6
Male	24.1	17.6	26.3	25.1	44.4
**Age group**					
16-29	100.0	69.4	45.7	49.4	17.6
30-59	0.0	24.6	52.3	49.3	63.4
60 or more	0.0	6.0	2.1	1.3	19.0
**Education level**					
Primary or less	17.2	19.9	28.4	22.3	51.4
Some secondary	75.9	45.4	42.6	40.9	29.6
At least completed secondary	6.9	34.7	29.1	36.8	19.0
**Professional status**					
Employed	0.0	7.1	12.3	14.0	7.9
Student	62.1	29.4	6.6	8.3	2.3
Looking for work	13.8	26.8	36.1	39.1	18.1
Other inactive	24.1	36.7	45.0	38.7	71.8
**Marital status**					
Never been married	100.0	87.3	84.8	88.8	70.4
Engaged	0.0	2.2	6.1	5.2	5.1
Married	0.0	8.8	6.8	4.8	17.1
Divorced / Separated / Windowed	0.0	1.7	2.3	1.2	7.4
**Household assets (wealth)**					
Low	17.2	27.2	29.9	28.4	33.3
Middle	55.2	45.2	43.7	44.9	40.3
High	27.6	27.6	26.3	26.7	26.4
**Total**	**100%**	**100%**	**100%**	**100%**	**100%**

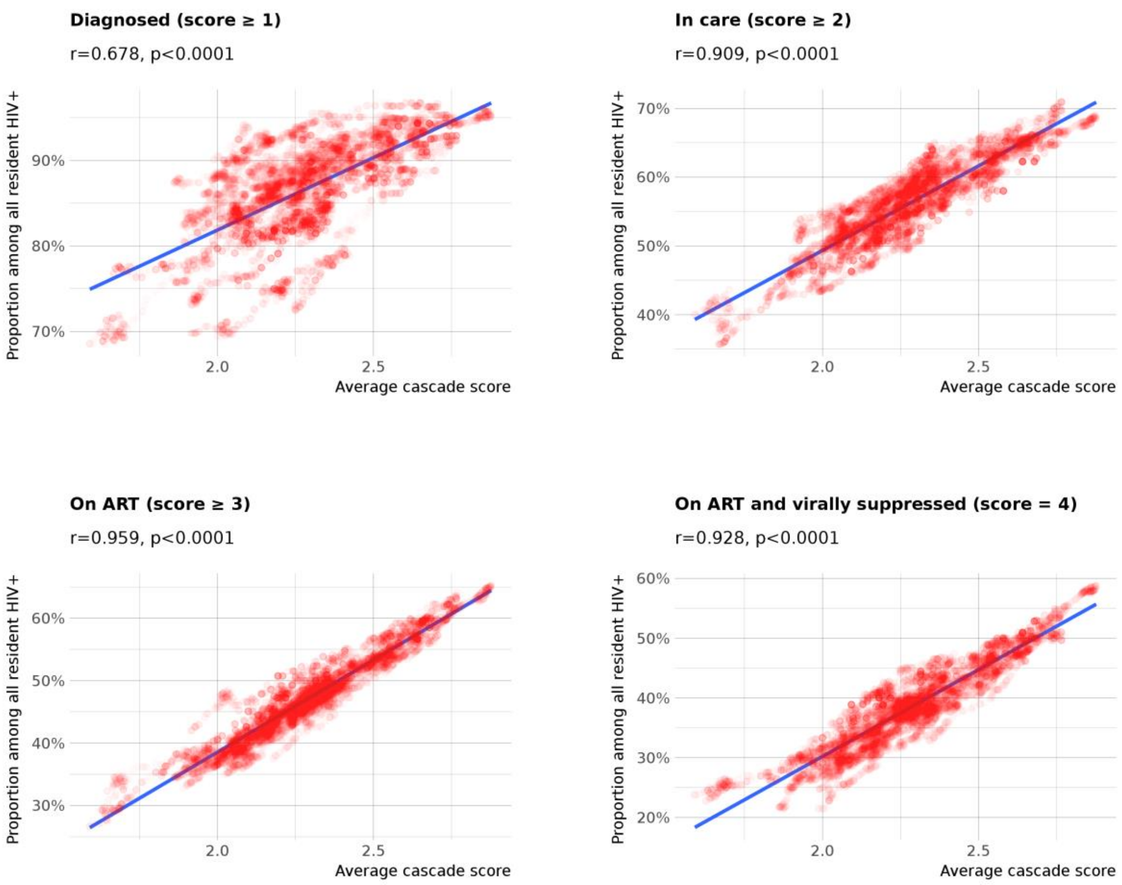

Figure S2. Correlation between the average cascade score and proportions of all resident PLWHIV being diagnosed, in care, on ART or virally suppressed at cluster level, ANRS 12249 TasP trial (2012-2016). Each dot represents a cluster at a given day. *r* is the Pearson correlation coefficient and *p* the significance of the statistical test evaluating if *r* differs from zero.

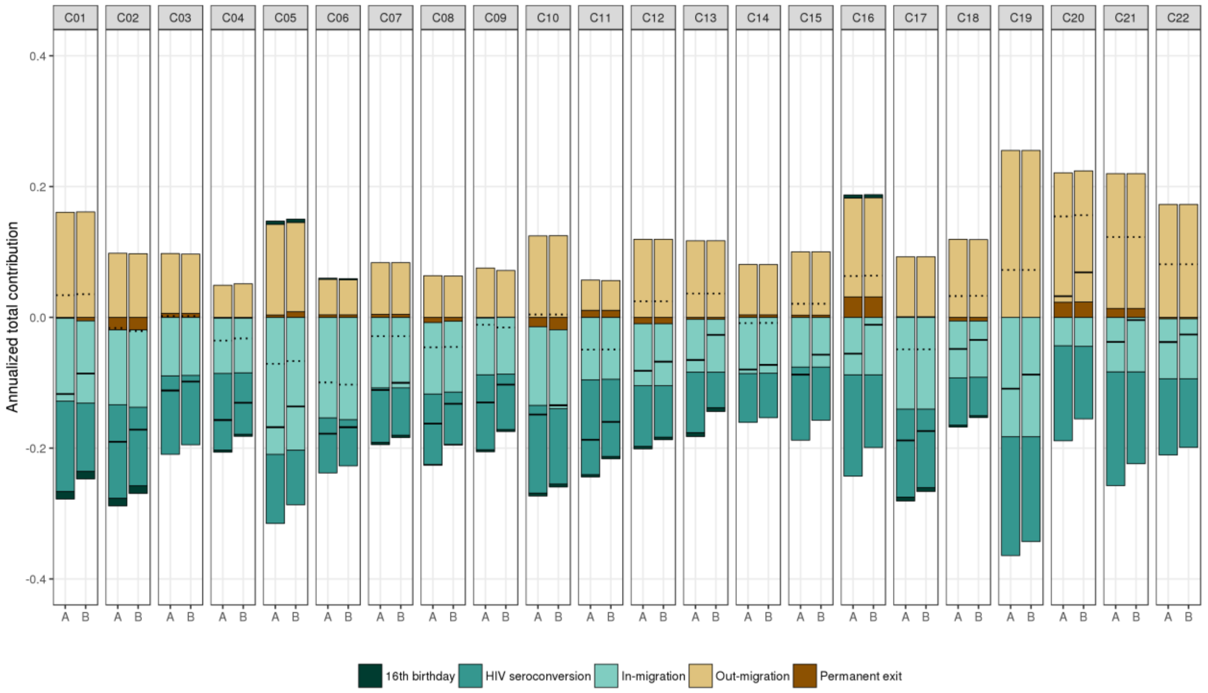

Figure S3. Sensitive analysis: comparison of annualized total contribution of population change on cluster average cascade score, by component of population change and per cluster, with (A) and without (B) imputation of potential unobserved seroconversions. In scenario B, individuals whose first observed HIV status was positive were considered already positive when they entered the trial cohort and individuals whose last observed HIV status was negative were considered still negative at the end of trial follow-up. Dotted lines indicate the sum of the total contribution of in- and out-migration. Black lines indicate the sum of total contribution of all events.
